# Durvalumab (MEDI 4736) in combination with extended neoadjuvant regimens in rectal cancer: a study protocol of a randomised phase II trial (PRIME-RT)

**DOI:** 10.1186/s13014-021-01888-1

**Published:** 2021-08-26

**Authors:** Catherine R. Hanna, Sean M. O’Cathail, Janet S. Graham, Mark Saunders, Leslie Samuel, Mark Harrison, Lynsey Devlin, Joanne Edwards, Daniel R. Gaya, Caroline A. Kelly, Liz-Anne Lewsley, Noori Maka, Paula Morrison, Louise Dinnett, Susan Dillon, Jacqueline Gourlay, Jonathan J. Platt, Fiona Thomson, Richard A. Adams, Campbell S. D. Roxburgh

**Affiliations:** 1 Cancer Research UK Glasgow Clinical Trials Unit, Beatson West of Scotland Cancer Centre, Institute of Cancer Sciences, University of Glasgow, Level 0, 1053 Great Western Road, Glasgow, G12 0YN UK; 2 Beatson West of Scotland Cancer Centre, Institute of Cancer Sciences, University of Glasgow, 1053 Great Western Road, Glasgow, G12 0YN UK; 3 Beatson West of Scotland Cancer Centre, 1053 Great Western Road, Glasgow, G12 0YN UK; 4 The Christie NHS Foundation Trust, Wilmslow Rd, Manchester, M20 4BX UK; 5 Aberdeen Royal Infirmary, Aberdeen, AB25 2ZN UK; 6 Mount Vernon Cancer Centre, Rickmansworth Rd, Northwood, HA6 2RN UK; 7 Institute of Cancer Sciences, University of Glasgow, Garscube Estate, Switchback Road, Bearsden, G61 1QH UK; 8 Gastroenterology Unit, Glasgow Royal Infirmary, NHS Greater Glasgow and Clyde, 4th Floor Walton Building, Castle Street, Glasgow, G4 0SF UK; 9 Queen Elizabeth University Hospital, NHS Greater Glasgow and Clyde, 1345 Govan Road, Glasgow, G51 4TF UK; 10 Snr Pharmacist Clinical Trials Oncology R&I, Research & Innovation, Dykebar Hospital, NHS Greater Glasgow & Clyde, Ward 11, Grahamston Road, Paisley, PA2 7DE UK; 11 Department of Radiology, NHS Greater Glasgow and Clyde, Glasgow Royal Infirmary, 84 Castle Street, Glasgow, G4 0SF UK; 12 Institute of Cancer Sciences, University of Glasgow, Glasgow, G61 1QH UK; 13 Centre for Trials Research Cardiff University Heath Park, Cardiff University and Velindre NHS Trust, Cardiff, UK; 14 Institute of Cancer Sciences, Glasgow Royal Infirmary, University of Glasgow, Room 2.57, Level 2, New Lister Building, Glasgow, G31 2ER UK

**Keywords:** Rectal, Neoplasm, Chemotherapy, Radiotherapy, Immune-oncology, Immunotherapy, Clinical trial

## Abstract

**Background:**

Advances in multi-modality treatment of locally advanced rectal cancer (LARC) have resulted in low local recurrence rates, but around 30% of patients will still die from distant metastatic disease. In parallel, there is increasing recognition that with radiotherapy and systemic treatment, some patients achieve a complete response and may avoid surgical resection, including in many cases, the need for a permanent stoma. Extended neoadjuvant regimes have emerged to address these concerns. The inclusion of immunotherapy in the neoadjuvant setting has the potential to further enhance this strategy by priming the local immune microenvironment and engaging the systemic immune response.

**Methods:**

PRIME-RT is a multi-centre, open label, phase II, randomised trial for patients with newly diagnosed LARC. Eligible patients will be randomised to receive either: short course radiotherapy (25 Gray in 5 fractions over one week) with concomitant durvalumab (1500 mg administered intravenously every 4 weeks), followed by FOLFOX (85 mg/m^2^ oxaliplatin, 350 mg folinic acid and 400 mg/m^2^ bolus 5-fluorouracil (5-FU) given on day 1 followed by 2400 mg/m^2^ 5-FU infusion over 46–48 h, all administered intravenously every 2 weeks), and durvalumab, or long course chemoradiotherapy (50 Gray to primary tumour in 25 fractions over 5 weeks with concomitant oral capecitabine 825 mg/m^2^ twice per day on days of radiotherapy) with durvalumab followed by FOLFOX and durvalumab. The primary endpoint is complete response rate in each arm. Secondary endpoints include treatment compliance, toxicity, safety, overall recurrence, proportion of patients with a permanent stoma, and survival. The study is translationally rich with collection of bio-specimens prior to, during, and following treatment in order to understand the molecular and immunological factors underpinning treatment response. The trial opened and the first patient was recruited in January 2021. The main trial will recruit up to 42 patients with LARC and commence after completion of a safety run-in that will recruit at least six patients with LARC or metastatic disease.

**Discussion:**

PRIME-RT will explore if adding immunotherapy to neoadjuvant radiotherapy and chemotherapy for patients with LARC can prime the tumour microenvironment to improve complete response rates and stoma free survival. Sequential biopsies are a key component within the trial design that will provide new knowledge on how the tumour microenvironment changes at different time-points in response to multi-modality treatment. This expectation is that the trial will provide information to test this treatment within a large phase clinical trial.

*Trial registration*

Clinicaltrials.gov NCT04621370 (Registered 9th Nov 2020)

EudraCT number 2019-001471-36 (Registered 6th Nov 2020)

**Supplementary Information:**

The online version contains supplementary material available at 10.1186/s13014-021-01888-1.

## Background

Locally advanced rectal cancer (LARC), defined as stage II or stage III disease, constitutes up to one third of rectal tumours at diagnosis [1]. Treatment of LARC requires a multi-modality approach because surgical resection alone results in unacceptably high rates of local recurrence [2]. Radiotherapy is the main therapeutic modality currently used prior to surgery, delivered either as short course (5 × 5 Gray (Gy) over one week) (SCRT) or as long course radiotherapy with concurrent fluoropyrimidine chemotherapy (45–50 Gy over 5 weeks) (LCRT) [3, 4]. Leaving a ‘gap’ following radiotherapy allows for maturation of tumour regression and improves down-staging [5–7]. Increasingly, cytotoxic chemotherapy is being delivered in this gap, primarily to treat micro-metastatic disease and reduce distant recurrence [8, 9]. This strategy shows improved compliance and tolerability, and improved response rates compared to chemotherapy delivered in the adjuvant setting [10–12], even with intensification of the chemotherapy regimen [12].

Using this approach of sequential radiotherapy and chemotherapy prior to surgery, also known as total neoadjuvant treatment, some patients achieve a clinical complete response (cCR) to treatment and if monitored carefully, a select number of these good responders can avoid surgery altogether. The evaluation of cCR is usually performed at the end of treatment and defined as the absence of clinically detectable tumour using radiological evidence, clinical examination and endoscopic appearances [13]. Reports suggest that the proportion of patients achieving cCR after chemoradiotherapy in the order of around 15% [14, 15]. Pathological complete response (pCR) is confirmation of no viable tumour at resection for patients who do proceed to surgery [16]. Published series have demonstrated similar disease free survival (DFS) and overall survival (OS) for patients who have organ preservation after achieving a cCR compared to those who undergo surgery [17, 18], with 3 year colostomy free survival of up to 74% [18]. Unfortunately, approximately 20% of patients do not achieve any down-staging and, whilst the majority of patients have some response, most will still require surgery [15, 19, 20]. Given that only a minority of patients are achieving organ preservation with current treatments, intensification of neoadjuvant therapies is a primary research goal in rectal cancer [21].

There is a strong rationale for selecting immune checkpoint blockade as a candidate therapy for treatment intensification in this context. Previous use of immunotherapy in colorectal cancer has been mainly limited to the metastatic setting, where good responses are seen particularly in patients with deficiency in mismatch repair (MMR) proteins within the tumour [22–24]. Although MMR deficiency is only detected in a minority of patients with LARC, the neoadjuvant setting provides an opportunity to evaluate this treatment in both MMR proficient, as well as MMR deficient LARC. Specifically, DNA damaging treatments used in the neoadjuvant setting such as radiotherapy [25] and chemotherapy [26, 27] induce cellular damage and the resulting antigen exposure can incite lymphocytic responses. In this respect, radiotherapy and chemotherapy may ‘prime’ the tumour microenvironment, and when used in combination with immunotherapy may enhance this host anticancer immune response. In addition, recent evidence has suggested that moving immunotherapy earlier in the disease trajectory when tumour is in situ may improve response rates [28]. Given that immune checkpoint blockade acts systemically, by using immunotherapy for treatment of LARC there is also the potential to reduce distant micro-metastases. Indeed, this systemic effect of immunotherapy may be increased through the combination with radiotherapy in what is known as an abscopal effect [29].

The PRIME-RT trial will test the intensification of neoadjuvant treatment for patients with LARC using immune checkpoint blockade combined with radiotherapy and chemotherapy. The main aims of the PRIME-RT trial are:

- To test whether the addition of anti-PD1 immunotherapy to extended neoadjuvant regimens results in high rates of complete response that may enable a larger proportion of patients with locally advanced rectal cancer to have organ preservation
- To evaluate the priming effect of adding chemotherapy and immune checkpoint blockade to patients receiving SCRT and those treated with LCRT.

## Methods/Design

**Fig. 1:**
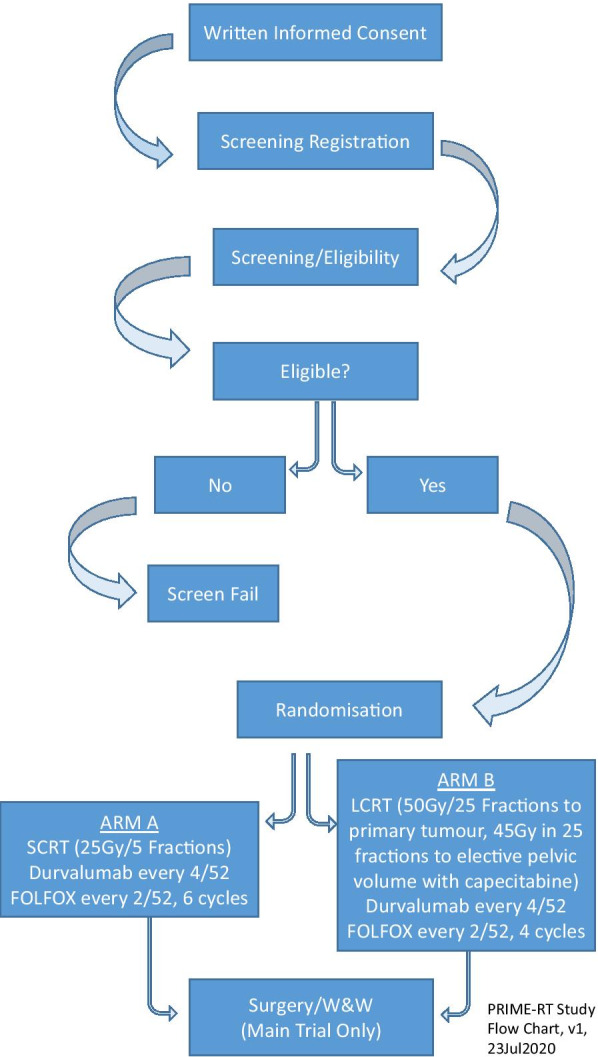
Process of recruitment of patients to PRIME-RT and random allocation of patients between treatment arms

PRIME-RT is an open label, multi-centre phase II randomised trial with 1:1 allocation between arm A and arm B (Fig. 1). The main trial will commence after completion of a safety run-in.

### Trial interventions

**Fig. 2:**
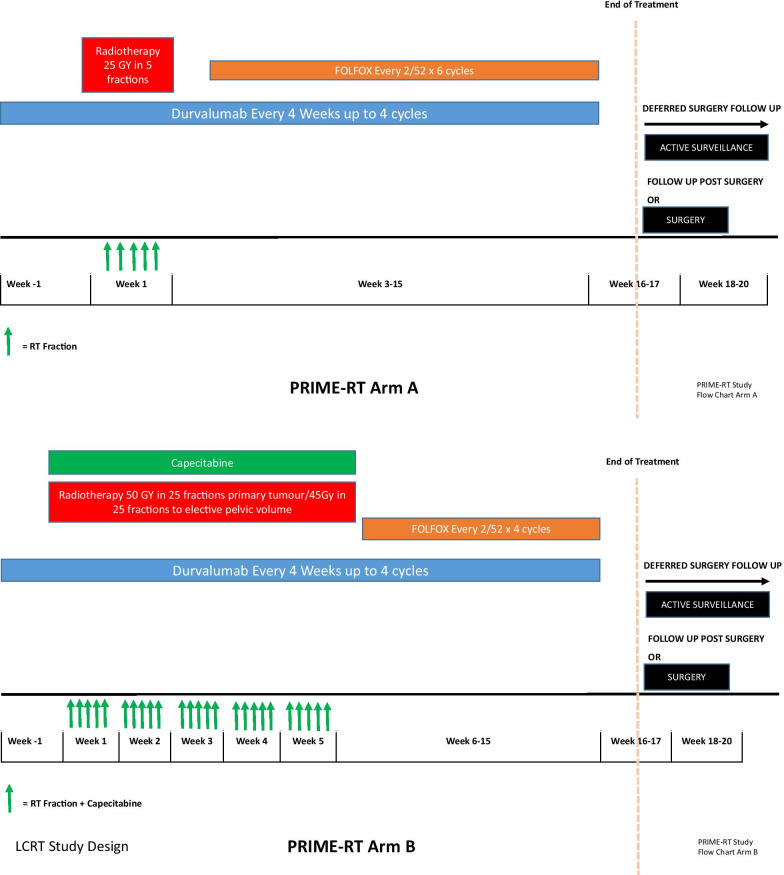
Treatment schema for Arm A and Arm B of PRIME-RT

The treatment schema for each arm of PRIME-RT is outlined in Fig. 2. Arm A consists of SCRT, followed by up to six cycles of FOLFOX chemotherapy. Patients also receive concurrent durvalumab (anti-PD-1 immune checkpoint inhibitor), commencing in the week prior to radiotherapy and continuing until completion of FOLFOX chemotherapy. In Arm B, patients receive LCRT followed by up to four cycles of FOLFOX chemotherapy, alongside concurrent durvalumab as for Arm A. LCRT consists of 50 Gy delivered to the primary tumour and 45 Gy to the elective pelvic nodes, delivered over 25 fractions with concomitant capecitabine chemotherapy 825 mg/m^2^ orally twice daily on the days of radiotherapy. Regarding LCRT, 45 Gy in 25 fractions has been the standard, accepted dose in the UK for pelvic rectal radiotherapy for many years and was used in a recent, large phase III rectal cancer trial (ARISTOTLE), which employed 3D conformal radiotherapy as the treatment technique [30]. There is evidence of a dose related response for rectal tumours [31], which has led to increased interest in using boost doses to areas of primary tumour. It is now more feasible to deliver boost doses safely and efficiently with the use of intensity modulated radiotherapy (IMRT). The dose levels used in PRIME-RT align with recently published United Kingdom (UK) consensus guidance, which has suggested using 45 Gy to elective pelvic nodes and a 50 Gy boost to primary tumour [32, 33].

For both arms, FOLFOX consists of a 400 mg/m^2^ bolus of fluorouracil over 10–15 min followed by a 2400 mg/m^2^ fluorouracil given as a continuous infusion over 46–48 h starting on day 1. Folinic acid 350 mg flat dose is administered over 2 h, in addition to oxaliplatin 85 mg/m^2^ over 2 h, both on day 1. Durvalumab is delivered as 1500 mg intravenously over 60 min every four weeks for up to four cycles in both arms. Radiotherapy treatment is delivered using inverse planned IMRT.

### Trial population

The target population for the main PRIME-RT trial consists of adult (≥ 18 years), fit (ECOG Performance Status 0–1) patients with a LARC which can be encompassed within a radical radiotherapy field. One of the following disease related features must be present on magnetic resonance imaging (MRI) assessment: cT3b+, EMVI positive, primary tumour or morphologically malignant lymph node at 2 mm or less from the mesorectal fascia or beyond the mesorectal fascia or a low rectal tumour requiring abdomino-perineal excision (APE). Patients are not permitted to enter the trial if they have any degree of dihydropyrimidine dehydrogenase deficiency, have received previous pelvic radiotherapy or have a history of inflammatory bowel disease or active autoimmune disease. Systemic steroid therapy or any other form of immunosuppressive medication within 14 days prior to the first dose of trial treatment is not permitted.

The trial criteria for the safety run-in is identical to the main trial except that patients are permitted to have metastatic disease with a primary rectal tumour in situ and to have received prior chemotherapy, but not immunotherapy, for the current malignancy. Additional File 1 contains a full list of inclusion and exclusion criteria. Patients for both parts of the trial will be recruited in the hospital setting and identified as potential trial candidates via multi-disciplinary meetings. The trial opened on 15th January 2021 with the first patient recruited to the safety run-in cohort on 28th January 2021.

### Study objectives and end-points

The principal objective addressed in PRIME-RT is whether the addition of durvalumab and FOLFOX chemotherapy to radiotherapy in the neoadjuvant setting for patients with LARC improves response rates. Specifically, the primary endpoint is a measurement of the proportion of patients achieving either complete clinical or pathological response in each trial arm. Both clinical and pathological complete response are included to ensure that patients undergoing surgery, as well as those following an organ preservation strategy, will both contribute to assessment of the primary endpoint. Clinical response will be assessed using a combination of MRI, endoscopic and clinical examination as outlined in Additional File 2, with the European Society of Gastroenterology and Abdominal Radiology (ESGAR) template [34] used for MRI reporting.

Secondary objectives include evaluation of safety and tolerability of treatment, including quality of life and surgical morbidity. Quality of life will be assessed at baseline and months 3, 6, 12, 18, 24 and 30 using three validated questionnaires (European Organization for Research and Treatment of Cancer Quality of Life Core Questionnaire 30, the European Organization for Research and Treatment of Cancer Quality of Life Questionnaire-Colorectal Cancer 29 and the Euro Qol-5 dimensions 3 levels survey). Treatment efficacy will be measured using additional secondary endpoints such as the neoadjuvant rectal score (NAR), MRI-confirmed tumour regression or near tumour regression or any degree of down-staging. Longer-term outcomes such as overall relapse rates and survival will also be measured.

CD3 + T-lymphocyte concentration is the main translational end-point for the trial and will be used to investigate the extent of immune activation within rectal biospecimens before, during (week 2 and 6) and after treatment. Biopsy assessment of CD3 + will be determined in tumour tissues by immunohistochemistry and divided into two groupings as none/low vs moderate/high based on previous descriptions [35–37] and treatment will be considered to be effective if a moderate-high CD3 + infiltrate is seen in 40% of patients.

Exploratory objectives for the PRIME-RT trial will include evaluation of biomarkers of treatment resistance and response to immunotherapy-radiotherapy combinations, assessment of the molecular characteristics of rectal cancers in which an immune priming response is achieved, and an analysis of how these responses may differ with respect to MMR status. The schedule for collection of data for the purposes of assessing all trial end-points is detailed in Additional File 3.

### Trial design and analysis plan

Each arm is a single arm phase II study design with its own safety run-in [38] and there will be no comparative analyses between arms. Permutated block randomisation will be used for the safety-run in and for the main trial a minimisation algorithm incorporating a random component will be used to allocate patients (1:1) between the arm A and arm B. Stratification will be dependent on whether the tumour is deemed resectable via (a) APE or (b) a sphincter-preserving resection (low anterior resection).

The safety run-in is being performed to identify any significant overlapping toxicities that could potentially preclude curative surgery in the main trial. After three patients in each arm of the safety run-in have completed treatment and been followed up for 30 days, recruitment will be postponed to allow a Committee to review all adverse event data. If a toxicity signal is detected, a decision will be made on the appropriateness of de-escalating treatment depending on which part of the treatment is most likely to be responsible. An additional three patients may be added to each arm of the safety run-in depending on the toxicity observed. The criteria for cohort expansion are detailed in Additional File 1. An Independent Data Monitoring Committee will make the decision on whether the randomised phase II component of PRIME-RT will open.

### Analysis plan and sample size calculation

Efficacy analyses will be on an intention to treat basis and safety analyses will include all patients who start treatment. For the primary efficacy analysis, a one-sided 80% confidence interval for the proportion of patients in each arm who have a complete response will be calculated using the Clopper–Pearson [39] approach. In each arm, treatment will be considered effective if at least five patients have a complete response. This pre-defined end-point for both trial arms is based on a hypothesis that complete response rates at six months will exceed 30% compared to rates of approximately 15% for standard long course chemoradiotherapy (LCRT) [14, 15]. If at least five patients in an arm have a complete response rate (equating to a success rate of 23.8%, with a lower one-sided 80% confidence limit of 15.1%), the hypothesis that the true efficacy is ≤ 15% is rejected. If the true complete response rate is 30% (the hypothesised level of efficacy used for the primary endpoint calculation), 21 patients per arm provides 80% power to demonstrate that the one-sided 80% confidence interval (20% 1-sided level of statistical significance) for the complete response rate excludes 15% (the minimum level of efficacy to warrant further evaluation).

If both arms appear to be equally effective, the main secondary endpoint (occurrence of Grade 3–5 treatment-emergent and treatment-related adverse events) will be used to determine the most appropriate trial arm to take forward for further study. The null hypothesis is that 50% of patients will not experience any Grade 3–5 toxicity, with an alternative hypothesis that 78% of patients will not experience any Grade 3–5 toxicity. If at least 14 participants in an arm experience no Grade 3–5 toxicity, (equating to a no toxicity rate of 66.6%, with a lower one-sided 90% confidence limit above 50%), the null hypothesis (that the true “no Grade 3–5 toxicity” rate is ≤ 50%) will be rejected in favour of the alternative hypothesis that the “no Grade 3–5 toxicity” rate is 78%. For the secondary endpoint calculation, 21 patients per arm provides 90% power to demonstrate that the one-sided 90% confidence interval (10% 1-sided level of statistical significant) for the “no Grade 3–5 toxicity” rate excludes 50%.

If both trial arms achieve a complete response rate of over 30% and demonstrate low toxicity as described above, a tiered consideration of other endpoints will be used to decide which treatment strategy to use moving forwards. In order of decreasing importance, these factors are; toxicity, absolute percentage of patients with complete response, surgical morbidity, the NAR score, local regrowth (36 months), overall survival (36 months), recurrence free survival (36 months), quality of life, percentage of patients with high grade CD3 + or a fold change in CD3 + compared to baseline.

## Discussion

PRIME-RT is investigating an important clinical question about the effectiveness, safety, and tolerability of intensifying LARC neoadjuvant treatment. The number of trials investigating novel strategies to improve responses to neoadjuvant treatment and to increase organ preservation rates that reported findings in 2020 [8, 12, 40, 41] demonstrates the intense interest in this approach within the academic and clinical community. A recent survey of patients with rectal cancer in Germany has confirmed that treatment intensification to improve organ preservation rates is also a concept favoured by patients, even at the expense of toxicity from multi-modality non-surgical treatment [42].

The PRIME-RT trial has the potential to have academic and wider impacts. The strong translational component to PRIME-RT provides a unique opportunity to analyse changes in the immune microenviroment in response to treatment and to track how this changes over time. This information will help to address the urgent need for biomarkers of response and resistance that are required by clinicians in order to tailor treatment to the patient in front of them in clinic. The radiotherapy protocol for PRIME-RT has been largely based on the novel, national IMRT rectal cancer guidance for the UK [32, 33]. Sites recruiting to PRIME-RT will have the opportunity to implement this new guidance in the context of the PRIME-RT trial and to have prospective peer review and quality assurance for their radiotherapy volumes and plans. If the effectiveness of either of the treatment strategies is proven, this signal will be tested in a larger phase trial. It is expected that patients within the trial may benefit from an increased rate of complete response and/or a reduction in distant recurrence compared to standard care; if tested within a larger trial this has the potential to be practice changing and improve outcomes on a larger scale. Lastly, this trial is only possible through collaboration between industry and academia. Building these relationships is an important endeavour to ensure investment in clinically relevant trials going forward. Two high profile consensus statements on novel drug radiotherapy combinations have highlighted five key messages [43, 44]: (1) the potential of combinations to improve outcomes, (2) importance of communication between industry, academia, regulatory agencies and patient advocates, (3) intelligent trial design, (4) validated endpoints and (5) novel approaches including immune-oncology combined with radiotherapy should be prioritised. PRIME-RT is part of new paradigm in the treatment of rectal cancer that satisfies all of these key aims.

Complete response rate has been chosen as the primary end-point for PRIME-RT because it is a clinically meaningful indicator of treatment efficacy and will allow comparison of outcomes from other early phase trials in this setting. It must be acknowledged however, the limitation of complete response as an outcome measure is that it is not a validated surrogate for longer term outcomes of disease free (DFS) or overall survival (OS) [45]. An important secondary endpoint in PRIME-RT is the NAR score, which will be measured for patients who undergo surgical resection. This score has been validated as a surrogate endpoint for predicting OS and DFS in clinical trials assessing neoadjuvant treatment for patients with locally advanced rectal cancer [45, 46]. Lastly, the main translational endpoint for PRIME-RT (CD3 + concentration) has been chosen based on the consistent observation that high density T cells are associated with improved survival independent of disease stage and mismatch repair status [47–49].

There are other trials in set-up and currently recruiting that are investigating the use of neoadjuvant immune checkpoint blockade for patients with LARC. Encouraging results have already been reported from the VOLTAGE trial in Japan where 5 cycles of nivolumab (anti-PDL1) post LCRT in 41 patients with cT3/4 rectal cancer resulted in pathological complete response rates of 30% in pMMR disease (11/37 patients) and 60% in dMMR disease (3/5 patients) [24]. In this study, subgroup analyses suggested improved responses were associated assessment of immunological biomarkers. PRIME-RT will build on the results of the VOLTAGE trial through measurement of MMR status and CD3 + concentration for all patients. In addition, PRIME-RT holds an important place in the current landscape because it is the only study currently investigating both SCRT and LCRT, which is important given it is not year clear which option offers optimal down-staging when combined sequentially with chemotherapy, or indeed which order [41, 50, 51].

A challenge of any early phase trial combining radiotherapy with novel therapies is the potential overlapping toxicity from the interventions used. Diarrhoea, in particular, is a concern in the PRIME-RT trial because, as well as being a symptom of some rectal cancers, it is a recognised adverse event from radiotherapy, chemotherapy, and immune checkpoint blockade. Reassuring results have recently been disseminated for the NRG-GI-002 trial [52] which investigated the use of pembrolizumab (anti-PD1) in combination with LCRT after 8 cycles of mFOLFOX6 chemotherapy. During LCRT in this trial, the rate of grade 3 + adverse events was 48.2% for the experimental arm versus 37.3% in the control arm. Nevertheless, actions have been taken to address concerns regarding toxicity from treatment in PRIME-RT. First, data from recently reported trials combining radiotherapy with chemotherapy alone have been used to power the toxicity analysis. Specifically, the expected range of Grade 3–5 adverse event rates is 22–50% for treatment without durvalumab, based on contemporaneous clinical trial data from the RAPIDO [8, 53, 54], Polish II [55], UNICANCER-PRODIGE 23 [12] and CAO/ARO/AIO-12 [46] clinical trials. The issue of overlapping toxicity has also be mitigated in PRIME-RT by the inclusion of the safety run-in cohort, which will test the safety and tolerability of the proposed treatment strategy in a small group of patients who are not receiving treatment with curative intent. If any safety signals are detected, adjustments will be made to the PRIME-RT treatment schedule with the aim of reducing this toxicity. Finally, a detailed algorithm has been provided within the trial protocol to direct clinicians of the pro-active measures that should be taken if a patient enrolled in PRIME-RT develops diarrhoea (Additional file 2).

## Conclusion

PRIME-RT is a translationally rich, phase II parallel arm trial treating patients with LARC with total neoadjuvant treatment in combination with immune checkpoint blockade. The trial will investigate the potential priming ability of radiotherapy and chemotherapy to enhance responses to immunotherapy with two different radiotherapy fractionation schedules and address several important unanswered clinical questions for patients with LARC.

## Trial Status

At the time of publication, PRIME-RT was open to recruitment in two UK centres. Trial recruitment began in January 2021, with the first patient enrolled on 27th January 2021, and the estimated recruitment end date is June 2022. The current PRIME-RT protocol is Version 2, 24th September 2020.

## Supplementary Information

Additional File 1. SPIRIT checklist.

Additional File 2 Complete response assessment table and Approach to management of diarrhoea.

Additional File 3. PRIME-RT schedule of assessments.

## Data Availability

Full trial protocol available by contacting Liz-Anne Lewsley (Liz-Anne.Lewsley@glasgow.ac.uk).

## Abbreviations

APE: Abdomino-perineal excision
CD3: Cluster of differentiation 3
DFS: Disease free survival
dMMR: Deficient mismatch repair
ECOG: Eastern Cooperative Oncology Group
EMVI: Extra mural vascular invasion
ESGAR: European Society of Gastroenterology and Abdominal Radiology
Gy: Gray
IMRT: Intensity modulated radiotherapy
LARC: Locally advanced rectal cancer
LCRT: Long course chemoradiotherapy
mg/m^2^: Milligrams per metre squared
MMR: Mismatch repair
MRI: Magnetic Resonance Imaging
NAR: Neoadjuvant rectal score
OS: Overall survival
PD-1: Programmed cell death-1
pMMR: Proficient mismatch repair
RT: Radiotherapy
SCRT: Short course radiotherapy
UK: United Kingdom
