# Correction to: Durvalumab (MEDI 4736) in combination with extended neoadjuvant regimens in rectal cancer: a study protocol of a randomised phase II trial (PRIME-RT)

**DOI:** 10.1186/s13014-021-01941-z

**Published:** 2021-12-02

**Authors:** Catherine R. Hanna, Sean M. O’Cathail, Janet S. Graham, Mark Saunders, Leslie Samuel, Mark Harrison, Lynsey Devlin, Joanne Edwards, Daniel R. Gaya, Caroline A. Kelly, Liz-Anne Lewsley, Noori Maka, Paula Morrison, Louise Dinnett, Susan Dillon, Jacqueline Gourlay, Jonathan J. Platt, Fiona Thomson, Richard A. Adams, Campbell S. D. Roxburgh

**Affiliations:** 1 Cancer Research UK Glasgow Clinical Trials Unit, Beatson West of Scotland Cancer Centre, Institute of Cancer Sciences, University of Glasgow, Level 0, 1053 Great Western Road, Glasgow, G12 0YN UK; 2 Beatson West of Scotland Cancer Centre, Institute of Cancer Sciences, University of Glasgow, 1053 Great Western Road, Glasgow, G12 0YN UK; 3 Beatson West of Scotland Cancer Centre, 1053 Great Western Road, Glasgow, G12 0YN UK; 4 The Christie NHS Foundation Trust, Wilmslow Rd, Manchester, M20 4BX UK; 5 Aberdeen Royal Infirmary, Aberdeen, AB25 2ZN UK; 6 Mount Vernon Cancer Centre, Rickmansworth Rd, Northwood, HA6 2RN UK; 7 Institute of Cancer Sciences, University of Glasgow, Garscube Estate, Switchback Road, Bearsden, G61 1QH UK; 8 Gastroenterology Unit, Glasgow Royal Infirmary, NHS Greater Glasgow and Clyde, 4th Floor Walton Building, Castle Street, Glasgow, G4 0SF UK; 9 Queen Elizabeth University Hospital, NHS Greater Glasgow and Clyde, 1345 Govan Road, Glasgow, G51 4TF UK; 10 Snr Pharmacist Clinical Trials Oncology R&I, Research and Innovation, Dykebar Hospital, NHS Greater Glasgow and Clyde, Ward 11, Grahamston Road, Paisley, PA2 7DE UK; 11 Department of Radiology, NHS Greater Glasgow and Clyde, Glasgow Royal Infirmary, 84 Castle Street, Glasgow, G4 0SF UK; 12 Institute of Cancer Sciences, University of Glasgow, Glasgow, G61 1QH UK; 13 Centre for Trials Research Cardiff University Heath Park, Cardiff University and Velindre NHS Trust, Cardiff, UK; 14 Institute of Cancer Sciences, Glasgow Royal Infirmary, University of Glasgow, Room 2.57, Level 2, New Lister Building, Glasgow, G31 2ER UK

## Correction to: Radiat Oncol (2021) 16:163. https://doi.org/10.1186/s13014-021-01888-1

After publication of this article [1], the authors reported that the Funding information section was incomplete. The following should have been added: “This work was supported by CRUK (Grant Number C61974/A2429).”

The original article [1] has been updated.
