# Supplementary material for: Durvalumab (MEDI 4736) in combination with extended neoadjuvant regimens in rectal cancer: a study protocol of a randomised phase II trial (PRIME-RT)
Source: Radiat Oncol. 2021 Aug 26;16:163. doi: 10.1186/s13014-021-01888-1 (PMC8393812; doi:10.1186/s13014-021-01888-1)
Supplement: Supplementary file 2 — Complete response assessment table and Approach to management of diarrhoea. [file 13014_2021_1888_MOESM2_ESM.docx]

|  | **Complete Response** | **Near Complete Response** | **Incomplete Response** |
| --- | --- | --- | --- |
| **Rectal Examination** | Normal | Smooth induration or minor mucosal abnormalities | Palpable tumour nodules |
| **Endoscopic Appearances** | Flat white scar Telangiectasia  No Ulcer  No nodularity | Irregular mucosa  Small mucosal nodules or minor mucosal abnormality  Superficial ulceration  Mild persisting erythema of the scar | Viable tumour |
| **MRI Appearances** | No residual tumour mass, normalised rectal wall, minor fibrosis, no/minimal restricted diffusion, no nodes or nodes <5mm short axis, no EMVI, no residual mesorectal tumour deposits | No residual tumour mass, fribrotic wall thickening, minimal/no focal restricted diffusion, no nodes or nodes <5mm, no EMVI, no residual mesorectal tumour deposits | Residual tumour mass present and/or focal high signal on DWI, nodes >= 5mm short axis, EMVI, residual mesorectal tumour deposits |

**Definition of complete clinical response. (Adapted from Smith JJ BMC Cancer 2015)(**[**57**](#_ENREF_57)**)**

Grade 1 diarrhoea

- Check baseline bloods (FBC, CRP, U&E, LFT, TFT)
- Check temperature and heart rate
- Send stool culture (MC&S and C.Difficile)
- Avoid high fibre diet
- Consider discussion with gastroenterologist
- When symptoms or evaluation indicate a perforation is suspected (such as sepsis, peritoneal signs, and ileus), a surgical consultation should be sought immediately

Grade 3/4 diarrhoea

Grade 2 diarrhoea

- Check baseline bloods (FBC, CRP, U&E, LFT, TFT)
- Check temperature and heart rate
- Send stool culture (MC&S and C.Difficile)
- Stool chart
- Avoid high fibre diet
- Discussion with gastroenterologist
- Consider flexible sigmoidoscopy
- Consider AXR if symptoms or signs of colitis
- When symptoms or evaluation indicate a perforation is suspected, a surgical consultation should be sought immediately
- Admission to hospital
- Urgent discussion with gastroenterologist re flexible sigmoidoscopy
- Stool chart
- Perform AXR
- Perform CT abdomen if symptoms of persistent pain, signs of peritonism or fever
- Recommend check TTG
- Check baseline bloods (FBC, CRP, U&E, LFT, TFT)
- Check temperature and heart rate
- Perform stool culture (MC&S and C.Difficile)
- Avoid high fibre diet
- Consider screen for TB (Quanti-FERON TB Gold), HIV, Hepatitis B and C (required as work-up for potential infliximab therapy to treat immunotherapy induced colitis)
- When symptoms or evaluation indicate a perforation is suspected, a surgical consultation should be sought immediately

**PRIME-RT management algorithm for diarrhoea.**
