# Supplementary material for: Durvalumab (MEDI 4736) in combination with extended neoadjuvant regimens in rectal cancer: a study protocol of a randomised phase II trial (PRIME-RT)
Source: Radiat Oncol. 2021 Aug 26;16:163. doi: 10.1186/s13014-021-01888-1 (PMC8393812; doi:10.1186/s13014-021-01888-1)
Supplement: Supplementary file 3 — PRIME-RT schedule of assessments. [file 13014_2021_1888_MOESM3_ESM.docx]

| ^1^Medical history including confirmation of histological diagnosis, prior treatment, concomitant disease and concomitant treatments, including review of steroid use |
| --- |
| ^2^Durvalumab can be given any time in the week prior to start of radiotherapy |
| ^3^Pregnancy Test - Human chorionic gonadotrophic (HGC) results must be obtained and reviewed before the first dose of IMP is administered for women of child bearing potential. A woman is considered of childbearing potential (WOCBP), i.e. fertile, following menarche and until becoming post-menopausal unless permanently sterile. Permanent sterilisation methods include hysterectomy, bilateral salpingectomy and bilateral oophorectomy. A postmenopausal state is defined as no menses for 12 months without an alternative medical cause. **This must be performed a maximum of 7 days before administration of study drugs**. |
| ^4^Physical Examination at baseline then symptom directed physical examination if clinically indicated |
| ^5^Vital signs - including temperature, seated blood pressure and pulse rate |
| ^6^Haematology - Full Blood Count (including white blood cells (WBC) with differential count (neutrophils, platelets and coagulation (INR or Prothrombin Time (PT), aPTT, Fibrinogen) |
| ^7^Biochemistry – amylase, sodium, potassium, adjusted calcium, phosphate, urea, creatinine, calculated creatinine clearance, total protein, albumin, alkaline phosphatase (alk phos), bilirubin, alanine aminotransferase (ALT) or aspartate aminotransferase (AST), magnesium, cortisol, thyroid function tests, glucose. NB TFTs, Cortisol and glucose are only required on weeks when durvalumab is being delivered |
| ^8^Toxicity assessment including acute, subacute and late neurotoxicity (assessed using CTCAE v5.0). Please note that Adverse Events relating to trial procedures should be collected from consent. Please see Section 9.1.10 for further information |
| ^9^Post surgery complications - Clavien-Dindo Classification |
| ^10^MRI to be done either when patients have completed all treatment or at week 15-18 post day 1 radiotherapy if treatment stopped early |
| 11Flexible sigmoidoscopy plus biopsy should be done at baseline, 2 weeks following day 1 of radiotherapy and 6 weeks following day 1 of radiotherapy and atend of treatment. Flexibility of +/- 7 days either side of week 2 and week 6 permitted. Flexible sigmoidoscopy and biopsy at six weeks following radiotherapy is encouraged but not mandatory. If it is not possible to do a biopsy at week 6, flexible sigmoidoscopy alone is still encouraged. |
| ^12^ Patients with complete clinical response will enter the deferral of surgery protocol. Patients with near complete responses could be re-assessed in 4-6 weeks by endoscopy and only if improving appearances (e.g. epithelialising ulcer or continued regression of nodularity), a deferral of surgery path would be followed. Patients entering the deferral of surgery pathway should have flexible sigmoidoscopies and pelvic MRI scans at months 3, 6, 9, 12, 18, 24 and 30 (unless patient undergoing surgery); CT scan chest, abdomen and pelvis at 12, 24 and 36 months. |
| ^13^ QoLs should be performed at baseline and then at 3, 6, 12, 18, 24 and 30 months. QoLs should be completed prior to the patient being reviewed by the PI/designee and any concerns raised discussed. |
| ^14^ There is no time limit on when DPD can be tested prior to study entry |
